# Association of sleep characteristics with renal function in menopausal women without recognized chronic kidney disease

**DOI:** 10.3389/fpsyt.2022.1024245

**Published:** 2022-11-09

**Authors:** Jianqian Tong, Changbin Li, Jiangshan Hu, Yincheng Teng, Yang Zhou, Minfang Tao

**Affiliations:** ^1^Department of Gynecology and Obstetrics, Shanghai Jiao Tong University of Medicine Affiliated Sixth People’s Hospital, Shanghai, China; ^2^Department of Gynecology and Obstetrics, Shanghai Eighth People’s Hospital, Affiliated to Jiangsu University, Shanghai, China

**Keywords:** sleep characteristics, cystatin C, renal function, menopause, menopausal hormone therapy (HT)

## Abstract

**Objective:**

To delineate the association between sleep characteristics and renal function in peri-post menopause free of Chronic kidney disease (CKD) as well as cardiometabolic and hormone indicators.

**Methods:**

Cross-sectional data from a total of 823 Han-Chinese women aged 40–67 years who visited the Menopause Clinic in the Shanghai Sixth People’s Hospital from November 2011 to November 2020 were analyzed through the Pittsburgh Sleep Quality Index (PSQI) and serum cystatin C (Cys-C). Logistic regression models were used to assess the association between cumulative/each sleep parameter and renal function after adjusting for cardiometabolic variables.

**Results:**

After confounding factors, we identified that poor perceived sleep quality, shorter sleep duration (<6 h), low sleep efficiency (<75%), delayed sleep latency and worse sleep disturbance elevated more than doubled the odds ratio for declining renal function (≥0.91 mg/dL, the highest Cys-C) in postmenopause in a graded fashion. Meanwhile, multiple logistic regression analysis revealed that sleep disorder (PSQI ≥ 8), late postmenopause, highest quartile independently increased the odds ratio for declining renal function (OR 2.007, 95% CI: 1.408–2.861, OR = 3.287, 95%CI: 3.425–8.889, OR = 2.345, 95% CI: 1.310–4.199, respectively), while participants with menopausal hormone replacement (MHT) lower the odds of declining renal function (OR = 0.486, 95% CI: 0.324–0.728).

**Conclusion:**

The findings proposed that maintaining good sleep quality should be attached great importance to postmenopausal women, which provides clinical evidence for the feasible early detection and effective prevention such as MHT of renal disease progression in postmenopausal women.

## Introduction

Chronic kidney disease (CKD) is determined as ongoing deterioration of renal function manifested by decreased glomerular filtration rate (GFR), leading to an increasing risk of hospitalization and mortality (1), and thus results in substantial health economic burden globally (2). As an endocrine organ, the kidney serves as the main target for hormone action (3). Many studies have revealed that menopause was supported to be associated with a higher risk of developing CKD due to diminished ovarian hormones (3–5). Therefore, identification of potential patients in menopause is paramount for the initiation of effective therapies to slow or delay disease progression.

Although estimated GFR (eGFR), based on the measurement of serum creatinine, is the most commonly used method to evaluate renal function, the change of creatinine is not significant in the early stages of renal impairment and influenced by muscle mass and body weight (6), which could result in less sensitivity of eGFR measurements (7). While Serum cystatin C (Cys-C), constantly secreted from all nucleated cells, has been purported as a more sensitive and specific biomarker than serum creatinine (8, 9). Therefore, Cys-C may be highly applicable as an early marker of preclinical renal disease. In addition, it serves as a much better-diagnostic tool for kidney function independent of age, sex, inflammation, liver disease, diet, and individual constitution and muscle mass (10, 11).

Menopause is a critical physiological stage of women’s life with various complaints. Besides vasomotor symptoms, sleep disorder is another marker of menopause (12). Women who experience poor sleep are more vulnerable to diseases, which is of great concern for women’s life quality and long-term health. In addition, a review proposed that OSAHS (obstructive sleep apnea-hypopnea syndrome) may contribute to CKD development either indirectly through its influences on diabetes, obesity and hypertension, or directly through the sympathetic nervous system and renin-angiotensin-aldosterone system (13). However, literature on the relationship between sleep disorder and clinically latent renal disease in menopause is scant.

As the burden of CKD in women after menopause is increasing, there is an emerging need for menopausal women-based research designed to disentangle the interactions between sleep disorder and renal function. In this study, we aim to investigate the association between sleep characteristics evaluated by the Pittsburgh Sleep Quality Index (PSQI) and serum Cys-C in terms of menopause, to identify the potential predicting value of sleep disorder for preclinical kidney disease among Chinese women without CKD in different menopausal status.

## Materials and methods

### Study design and participants

This cross-sectional study enrolled participants who visited the Menopause Clinic in the Shanghai Sixth People’s Hospital from November 2011 to November 2020. Han-Chinese woman aged 40–67 years were recruited. The study protocol was approved by the Ethics Committee of Shanghai Sixth People’s Hospital, and the study was performed in accordance with the approved guidelines. All the participants provided written informed consents after full explanation of the study. All study protocols were performed in accordance with the principles of the Declaration of Helsinki. Participants were excluded as follows: (1) suspected renal insufficiency, with an eGFR less than 60 mL/min/1.73 m^2^; (2) history of chronic nephritis, nephrotic syndrome, nephrectomy, polycystic kidney disease, organ or bone marrow transplant, immunosuppressive drugs for kidney disease in the past 6 months; (3) night work shifts and irregular sleep schedule; (4) menopausal hormone replacement (MHT) past users (women who reported past not current use for over previous 6 months); (5) current smoking (at least once per week for the previous 6 months); (6) progressive malignancy currently undergoing radiotherapy or chemotherapy; (7) with missing data. Ultimately, 823 participants were recruited in this study.

### General questionnaire

A general questionnaire (14–16) was used by well-trained investigators through face-to-face interview to collect sociodemographic information, including age, last menstrual period, education, marital status, employment status, income per month, years since menopause, MHT use (including estradiol + dydrogesterone, estradiol only, tibolone), history of chronic disease (hypertension, diabetes mellitus, CKD, metabolic syndrome, dyslipidemia, obesity, as well as medication use), lifestyle (i.e., smoking, alcohol consumption). Women who reported current use ≥ 6 months were classified as current MHT users, while women who had never taken were classified as never users. On the basis of the Stages of Reproductive Aging Workshop (STRAW + 10) (17), participants were categorized into peri-menopausal group (consecutive irregularities over 7 days of menstrual cycle), early postmenopausal group (absence of menstrual periods for 1–5 years) and late postmenopausal group (absence of menstrual periods for 5 years or more) (14).

### Anthropometric and lab parameters

Height (cm) and weight (kg) were recorded and used to computed Body mass index BMI (kg/m^2^). Blood pressure was measured with the average of the 3 readings after 5-min sitting 0.19 Hypertension was defined as systolic blood pressure (SBP) ≥ 140 mm Hg, diastolic blood pressure (DBP) ≥ 90 mm Hg or use of antihypertensive medications (16).

After an overnight fast for at least 10 h, venous blood samples were collected for all study participants for biochemical measurements analysis. Serum Cys-C, creatine, total cholesterol (TC), triglyceride (TG), low-density lipoprotein cholesterol (LDL-C), high-density lipoprotein cholesterol (HDL-C) and creatinine were measured using an automated AU-5800 analyzer (Beckman Coulter, Brea, CA, USA). Fasting plasma glucose (FPG) was measured with the glucose oxidase method using an automated AU-5800 analyzer (Beckman Coulter, Brea, CA, USA). Female sex hormone levels were evaluated by chemiluminescence (Cobas E601; Roche, Basel, Switzerland). The sensitivity for follicle-stimulating hormone (FSH) detection was 0.100 mIU/mL, and the range of measurement was 0.100–200.0 mIU/mL; for estradiol (E2), the sensitivity and range of measurement was 5 pg/mL and 5–3,000 pg/mL, respectively. Intra- and inter-assay coefficients of variation were always <5% for FSH and E2.

### Definitions of study outcomes

Dyslipidemia was defined as previous diagnosis or meeting any of the following criteria: (1) TC ≥ 6.22 mmol/L; (2) TG ≥ 2.26 mmol/L; (3) LDL ≥ 4.14 mmol/L; (4) HDL<1.05 mmol/L (18). Hypertension and diabetes were diagnosed based on self-reported previous diagnosis, or by criteria ≥ 140/90 mmHg and FPG ≥ 7 mmol/L (18, 19). BMI ≥ 28 kg/m^2^ was regarded as obesity. Definition of metabolic syndrome (MetS) was defined as the presence of two or more of the following components (20): (1) obesity (waist-hip-ratio > 0.85 and/or BMI ≥ 28 kg/m^2^); (2) TG ≥ 1.7 mmol/L; (3) HDL-c < 1.05 mmol/L; (4) blood pressure ≥ 140/90 mmHg or current use of antihypertensive medications; (5) FBG ≥ 7.0 mmol/L. CKD was defined as eGFR < 60 (mL/min/1.73 m^2^), which was calculated by recently revised CKD Epidemiology Collaboration (CKD-EPI) equation for the Chinese population (21).

### Assessment of sleep quality

The validated Chinese version Pittsburgh Sleep Quality Index (PSQI) (22) was used to evaluate sleep quality over the past month for the participants. In brief, 18 items, including in the PSQI, were used to weigh scores based on the following 7 subscales: subjective sleep quality, sleep latency, sleep duration, sleep efficiency, sleep disturbance, use of sleeping medications, and daytime dysfunction. Each parameter ranges from 0 to 3 scale. Subjective sleep quality was categorized into 0 to 3 scores, corresponding to very good, good, poor, very poor, sleep duration into >7 h, 6–7 h, 5–6 h, <5 h, sleep efficiency into>85%, 75–85%, 65–75%, <65%, sleep use of medication into none, <1 time/week, 1–2 times/week, ≥ 3 times/week. The total PSQI ranged from 0 to 21, with a higher score indicating worse sleep quality. A PSQI score of 8 or higher was indicative of sleep disorder (14, 23), which has been recommended in Chinese clinical practice and research.

### Statistical analysis

All the variables were tested for normal distribution by Kolmogorov-Smirnov test. Levene’s test of homogeneity of variance were further performed. They were depicted as means ± standard deviation (SD) or number (%). One-way ANOVA (normal distributions), the Kruskal Wallis H-test (skewed continuous variables) and χ2 test (categorical variables) were carried out to compare the differences among the four groups on the quartiles of serum Cys-C levels (Q1:<0.70 mg/dL, Q2:0.71–0.72 mg/dL, Q3:0.73–0.90 mg/dL, Q4: ≥ 0.91 mg/dL). We defined Cys-C with 0.91 mg/dL (Q4, the highest quartile group) as declining renal function. The multivariate logistic regression analyses were performed to examine independent determinants for Q4. In addition, the association between each sleep parameter and Q4 was computed by logistic regression analysis. A two-sided *p* < 0.05 was considered to be a significant difference. Logistic regression model was assessed by the Hosmer-Lemeshow test. All statistical analyses were performed using SPSS 22.0 (IBM Corporation, Armonk, NY, USA).

## Results

### Characteristics of the study participants based on cystatin-C quartiles

In a total of 823 eligible subjects, the baseline characteristics among quartile groups divided by the serum Cys-C: (Q1: <0.70 mg/dL, Q2: 0.71–0.72 mg/dL, Q3: 0.73–0.90 mg/dL, Q4: ≥ 0.91 mg/dL) were presented in Table 1. The prevalence of sleep disorder (PSQI ≥ 8) was 38.6% in our study.

**TABLE 1:** Characteristics of the study participants distributed by quartile of cystatin C.

Variables	Q1	Q2	Q3	Q4	Total	*p*-value
	<0.70 mg/dL	0.71–0.72 mg/dL	0.73–0.90 mg/dL	≥0.91 mg/dL		
Age (years)	50.15 ± 5.04	51.18 ± 5.09	51.56 ± 4.67	52.58 ± 4.56	51.37 ± 4.91	<0.001
Height (cm)	159.7 ± 4.99	160.43 ± 4.5	160.59 ± 4.97	160.8 ± 5.18	160.38 ± 4.93	0.116
Weight (Kg)	55.51 ± 7.33	57.68 ± 7.9	57.94 ± 7.01	58.76 ± 7.99	57.47 ± 7.65	<0.001
BMI (Kg/m^2^)	55.51 ± 7.33	57.68 ± 7.9	57.94 ± 7.01	58.76 ± 7.99	57.47 ± 7.65	0.004
SBP (mmHg)	119.13 ± 14.99	118.15 ± 13.83	120.52 ± 15.62	120.75 ± 17.98	119.64 ± 15.69	0.289
DBP (mmHg)	72.75 ± 10.44	72.9 ± 9.62	74.32 ± 9.30	75.17 ± 10.16	73.79 ± 9.93	0.036
FPG (mmol/L)	5.3 ± 0.72	5.44 ± 1.06	5.37 ± 0.76	5.42 ± 0.86	5.38 ± 0.86	0.376
Tg (mmol/L)	1.27 ± 0.94	1.22 ± 0.72	1.23 ± 0.77	1.29 ± 0.95	1.25 ± 0.85	0.812
TC (mmol/L)	5.32 ± 0.99	5.16 ± 1.01	5.2 ± 1.07	5.1 ± 1.02	5.2 ± 1.02	0.154
HDL-C (mmol/L)	1.62 ± 0.5	1.52 ± 0.59	1.44 ± 0.4	1.5 ± 0.55	1.52 ± 0.52	0.005
LDL-C (mmol/L)	3.1 ± 0.82	3.1 ± 0.96	3.13 ± 0.89	3.06 ± 0.81	3.1 ± 0.87	0.873
E2 (pg/mL)	68.99 ± 98.61	60.59 ± 79.23	51.04 ± 71.6	63.44 ± 131.86	61.05 ± 98.29	0.309
FSH (mIU/mL)	59.89 ± 31.89	63.62 ± 31.27	67.43 ± 28.95	70.69 ± 30.27	65.41 ± 30.83	0.002
Cystatin C (mg/L)	0.58 ± 0.07	0.72 ± 0.01	0.83 ± 0.04	1.05 ± 0.15	0.79 ± 0.19	<0.001
Creatine	53.74 ± 8.85	56.75 ± 7.03	58.28 ± 8.06	61.32 ± 11.18	57.52 ± 9.32	<0.001
eGFR (mL/min/1.73 m^2^)	129.61 ± 24.81	119.86 ± 17.73	116.89 ± 21.09	111.69 ± 26.56	119.52 ± 23.70	<0.001
PSQI total score	7.29 ± 4.72	7.15 ± 4.39	8.25 ± 4.97	10.02 ± 4.87	8.18 ± 4.87	<0.001
PSQI ≥ 8	81 (39.13%)	85 (41.46%)	99 (48.53%)	135 (65.22%)	400 (38.60%)	<0.001
Years since menopause	1.95 ± 2.89	3.02 ± 4.68	3.03 ± 4.25	3.53 ± 4.86	2.89 ± 4.28	0.002
**Menopausal status, *n* (%)**						<0.001
Perimenopause	104 (50.24%)	78 (38.05%)	62 (30.39%)	52 (25.12%)	296 (35.97%)	
Early postmenopause	73 (35.27%)	77 (37.56%)	88 (43.14%)	104 (50.24%)	342 (41.56%)	
Late postmenopause	30 (14.49%)	50 (24.39%)	54 (26.47%)	51 (24.64%)	185 (22.48%)	
**Marital status**						0.261
Married	199 (96.14%)	200 (97.56%)	200 (98.04%)	205 (99.03%)	804 (97.69%)	
Single/widowed	8 (3.86%)	5 (2.44%)	4 (1.96%)	2 (0.97%)	19 (2.31%)	
**Education, *n* (%)**						0.305
Junior or below	30 (14.49%)	38 (18.54%)	45 (22.06%)	38.48 (26.14%)	153 (18.59%)	
Senior high	74 (35.75%)	83 (40.49%)	80 (39.22%)	79.48 (25%)	316 (38.4%)	
College or above	103 (49.76%)	84 (40.98%)	79 (38.73%)	89.04 (24.86%)	354 (43.01%)	
**Employment status, *n* (%)**						0.051
Work	119 (57.49%)	116 (56.59%)	93 (45.59%)	94 (45.41%)	422 (51.28%)	
Departure	74 (35.75%)	78 (38.05%)	90 (44.12%)	106 (51.21%)	348 (42.28%)	
Retirement	14 (6.76%)	11 (5.37%)	21 (10.29%)	7 (3.38%)	53 (6.44%)	
**Income (RMB/month), *n* (%)**						0.052
<1,000	16 (7.73%)	20 (9.76%)	19 (9.31%)	6 (2.9%)	61 (7.41%)	
1,000–3,000	48 (23.19%)	58 (28.29%)	66 (32.35%)	82 (39.61%)	254 (30.86%)	
3,000–5,000	54 (26.09%)	68 (33.17%)	69 (33.82%)	68 (32.85%)	259 (31.47%)	
5,000–10,000	58 (28.02%)	34 (16.59%)	37 (18.14%)	37 (17.87%)	166 (20.17%)	
>10,000	31 (14.98%)	25 (12.2%)	13 (6.37%)	14 (6.76%)	83 (10.09%)	
Overweight/obesity, *n* (%)	23 (11.11%)	32 (15.61%)	31 (15.20%)	48 (23.19%)	136 (16.52%)	0.018
Hypertension	26 (12.08%)	26 (12.68%)	41 (20.10%)	65 (31.40%)	158 (19.20%)	<0.001
Diabetes	9 (4.35%)	15 (7.32%)	15 (7.35%)	26 (12.56%)	65 (7.90%)	0.019
Dyslipidemia, *n* (%)	69 (33.33%)	85 (41.46%)	78 (38.24%)	77 (37.2%)	309 (37.55%)	0.398
MetS, *n* (%)	10 (4.83%)	13 (6.34%)	23 (11.27%)	23 (11.11%)	69 (8.38%)	0.033
MHT use, *n* (%)	75 (36.23%)	99 (48.29%)	70 (34.31%)	51 (24.64%)	295 (35.84%)	<0.001

Two-sided *P* < 0.05 was considered significant.

eGFR, estimated glomerular filtration rate; BMI, body mass index; SBP, systolic blood pressure; FBG, fast blood glucose; TC, total cholesterol; TG, triglyceride; LDL-C, low-density lipoprotein cholesterol; HDL, high-density lipoprotein cholesterol; E2, estriol; FSH, follicle-stimulating hormone; MetS, metabolic syndrome; MHT, menopausal hormone replacement.

Interestingly, the prevalence of sleep disorder (PSQI ≥ 8) increased in a dose-dependent manner according to the serum of Cys-C: 39.13% (Q1) vs.41.46% (Q2) vs.48.53% (Q3) vs.65.22% (Q4) (*p*< 0.001). We also observed the growing value of FSH, years since menopause, age, BMI, DBP, creatine, while decreasing level of eGFR from Q1 to Q4 quartiles (*p* < 0.001). Moreover, there showed a decreasing incidence of MHT use, while an ascending incidence of early, late postmenopause (compared with perimenopause), as well as hypertension, diabetes mellitus with the increasing of Cys-C quartiles (*p* < 0.05). On the other hand, lipid profiles (including TC, TG, LDL-C, HDL-C), FBG, E2, SBP did not show significant trends across Cys-C quartiles. Furthermore, Table 2 showed score for sub-scales of PSQI based on Cys-C quartiles. There were significant differences in six sub-scales of PSQI (subjective sleep quality, sleep latency, sleep duration, sleep efficiency, sleep disturbance and daytime dysfunction) (All *p* < 0.05).

**TABLE 2:** Separate sleep parameter of PSQI by quartile of cystatin C.

PSQI items	Q1	Q2	Q3	Q4	Total	*p*-value
	<0.70 mg/dL	0.71–0.72 mg/dL	0.73–0.90 mg/dL	≥0.91 mg/dL		
**Subjective sleep quality**						<0.001
Very good	28 (13.53%)	31 (15.12%)	27 (13.24%)	16 (7.73%)	102 (12.39%)	
Good	123 (59.42%)	108 (52.68%)	109 (53.43%)	87 (42.03%)	427 (51.88%)	
Bad	38 (18.36%)	50 (24.39%)	46 (22.55%)	65 (31.40%)	199 (24.18%)	
Very bad	18 (8.70%)	16 (7.80%)	22 (10.78%)	39 (18.84%)	95 (11.54%)	
**Sleep latency (minutes)**						<0.001
≤15	85 (41.06%)	93 (45.37%)	83 (40.69%)	70 (33.82%)	331 (40.22%)	
16–30	74 (35.75%)	73 (35.61%)	63 (30.88%)	49 (23.67%)	259 (31.47%)	
31–60	29 (14.01%)	20 (9.76%)	31 (15.20%)	32 (15.46%)	112 (13.61%)	
>60	19 (9.18%)	19 (9.27%)	27 (13.24%)	56 (27.05%)	121 (14.70%)	
**Sleep duration (hours)**						<0.001
>7	60 (28.99%)	53 (25.85%)	47 (23.04%)	24 (11.59%)	184 (22.36%)	
6–7	63 (30.43%)	59 (28.78%)	53 (25.98%)	39 (18.84%)	214 (26%)	
5–6	57 (27.54%)	65 (31.71%)	71 (34.8%)	92 (44.44%)	285 (34.63%)	
<5	27 (13.04%)	28 (13.66%)	33 (16.18%)	52 (25.12%)	140 (17.01%)	
**Sleep efficiency(%)**						<0.001
>85	111 (54.68%)	107 (52.45%)	83 (41.09%)	63 (30.58%)	364 (44.66%)	
75–84	26 (12.81%)	32 (15.69%)	34 (16.83%)	25 (12.14%)	117 (14.36%)	
65–74	25 (12.32%)	34 (16.67%)	38 (18.81%)	48 (23.30%)	145 (17.79%)	
<65	41 (20.2%)	31 (15.2%)	47 (23.27%)	70 (33.98%)	189 (23.19%)	
**Sleep disturbance**						<0.001
None	47 (22.71%)	46 (22.44%)	29 (14.22%)	14 (6.76%)	136 (16.52%)	
<1/week	132 (63.77%)	128 (62.44%)	118 (57.84%)	117 (56.52%)	495 (60.15%)	
1–2/week	27 (13.04%)	31 (15.12%)	55 (26.96%)	75 (36.23%)	188 (22.84%)	
≥3/week	1 (0.48%)	0 (0.00%)	2 (0.98%)	1 (0.48%)	4 (0.49%)	
**Use of sleep medication**						0.171
None	173 (83.57%)	179 (87.32%)	169 (82.84%)	165 (79.71%)	686 (83.35%)	
<1/week	11 (5.31%)	10 (4.88%)	9 (4.41%)	9 (4.35%)	39 (4.74%)	
1–2/week	7 (3.38%)	2 (0.98%)	3 (1.47%)	11 (5.31%)	23 (2.79%)	
≥3/week	16 (7.73%)	14 (6.83%)	23 (11.27%)	22 (10.63%)	75 (9.11%)	
**Daytime dysfunction**						0.024
None	48 (23.19%)	58 (28.29%)	41 (20.1%)	38 (18.36%)	185 (22.48%)	
<1/week	69 (33.33%)	63 (30.73%)	71 (34.8%)	51 (24.64%)	254 (30.86%)	
1–2/week	53 (25.6%)	44 (21.46%)	42 (20.59%)	59 (28.5%)	198 (24.06%)	
≥3/week	37 (17.87%)	40 (19.51%)	50 (24.51%)	59 (28.5%)	186 (22.60%)	

### Independent determinant factors for declining renal function

**FIGURE 1:**
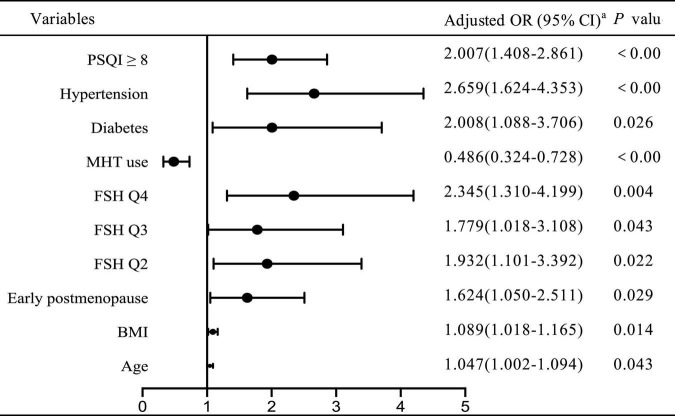
Adjusted odds ratio for declining renal function by logistic regression ^a^adjusted for age, BMI, menopausal status, years since menopause, SBP, FBG, lipid profiles, hypertension, diabetes, obesity, MetS. OR, odds ratio; CI, confidential interval.

To investigate the independent determinant factors for declining renal function, we conducted a multivariate logistic stepwise regression analysis. As shown in Figure 1, after adjusting for confounding covariates, including age, BMI, menopausal status, years since menopause, FBG, lipid profiles (TC, TG, LDL-C, HDL-C), SBP, hypertension, diabetes, obesity, MetS, we identified the independent risk factors for declining renal function as PSQI ≥ 8 [odds ratio (OR) 2.007, 95% confidential interval (CI): 1.408–2.861], hypertension (OR 2.659, 95% CI: 1.624–4.353), diabetes (OR 2.008, 95% CI: 1.008–3.706), early postmenopause (OR 1.624, 95% CI: 1.050–2.511), BMI (OR 1.089, 95% CI: 1.018–1.165), age (OR 1.047, 95% CI: 1.002–1.094), while MHT served as a protective role in renal function (OR = 0.486, 95% CI: 0.324–0.728). In addition, the regression analysis revealed that the ORs of declining renal function were twofold higher (OR = 2.345, 95% CI: 1.310–4.199, *p* < 0.001) in the highest FSH quartile compared to the lowest.

### Odds ratio of sleep disorder for declining renal function stratified by menopause status

**FIGURE 2:**
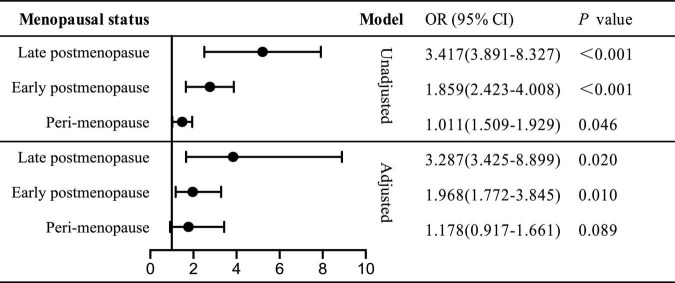
Odds ratio of PSQI ≥ 8 for declining renal function stratified by menopause status.

We further investigated the role of menopause in sleep disorder-renal function relation in unadjusted or multi-covariates adjusted model (Figure 2). Then the total subjects were divided into three groups: peri-, early post- and late post-menopause. However, in peri-menopause, the risk indicator of PSQI ≥ 8 for declining renal function vanished (OR = 1.178, 95% CI: 0.917–1.661, *p* = 0.089), while the ORs of declining renal function were twofold (OR = 1.968, 95% CI: 1.772–3.845) and threefold (OR = 3.287, 95% CI: 3.425–8.889) higher in early and late postmenopausal status, respectively. Thus, we put forward that in postmenopausal not perimenopause women, sleep disorder served as an independent determinant for declining renal function, while the odds ratio was higher in late postmenopause than in early one.

### Odds ratio of each sleep parameter of Pittsburgh Sleep Quality Index for declining renal function

**FIGURE 3:**
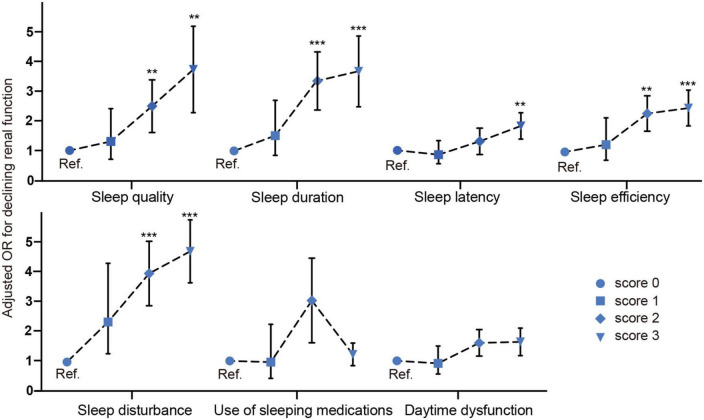
Odds ratio of each sleep parameter of PSQI for declining renal function. **p < 0.05, ***p < 0.001.

Next, we investigated the association between each sleep parameter and declining renal function. As shown in Figure 3, after multivariable adjustment of confounding factors, compared with >7 h (score 0), 5–6 h [score 2 OR 95% CI: 3.073 (1.824–5.178)] and <5 h [score 3 OR 95% CI: 3.295 (1.837–5.91), *p*<0.001] were associated with threefold higher odds ratio for declining renal function. Compared with sleep efficiency 85% (score 0), 65–75% [score 2 OR 95% CI: 2.145 (1.351–3.403)] and<65% [score 3 OR 95% CI: 2.338 (1.522–3.591), *p*<0.001] were associated with twofold higher odds for declining renal function.

The other significant sleep parameters were sleep quality [score 2 OR 95% CI: 2.179 (1.139–4.169), score 3 OR 95% CI: 3.165 (1.543–6.491), *p* < 0.05], sleep latency [score 3 OR 95% CI: 1.719 (1.117–2.647), *p* < 0.05], sleep disturbance [score 2 OR 95% CI: 4.047 (2.084–7.858), score 3 OR 95% CI: 4.233 (3.215–6.798), *p* < 0.05], sleep efficiency [score 2 OR 95% CI: 2.145 (1.351–3.403), score 3 OR 95% CI: 2.338 (1.522–3.591), *p* < 0.001]. In a summary, our fully adjusted model revealed that subjective poor sleep quality, shorter sleep duration (<6 h), low sleep efficiency (<75%) and longer sleep latency and higher sleep disturbance were independently associated with declining renal function in a graded fashion.

### Sleep disorder and menopausal hormone replacement on the prevalence of declining renal function

To investigate the role of MHT in sleep disorder-renal function relation, we then distinguished the participants by using MHT and sleep disorder to analyze the prevalence of declining kidney function (Cys-C ≥ 0.91 mg/dL, Q4) with different combinations of two-score based PSQI and MHT.

**FIGURE 4:**
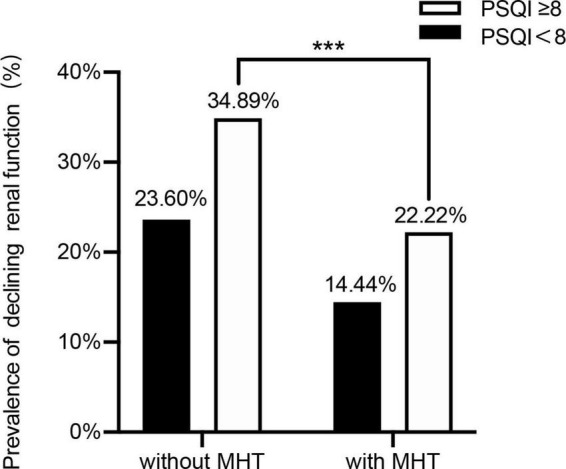
Sleep disorder and MHT on the prevalence of declining renal function. ***p < 0.001.

Compared with MHT non-users, the prevalence of sleep disorder for declining kidney function was significantly lower than in MHT user group (34.89 vs. 22.22%) (*p* < 0.05). On the whole, we found that the prevalence of declining renal function in sleep disorder was higher than without sleep disorder group, while MHT may lower the prevalence (Figure 4).

## Discussion

To our knowledge, this is the first study to document the relationship between sleep characteristics (total PSQI score as well as each sleep parameter) and latent renal function (estimated by serum Cys-C) in peri-post menopausal women without CKD in addition to cardiometabolic and hormone indicators. In our study, we found that sleep disorder (PSQI ≥ 8) served as a determinant risk factor for declining renal function (Cys-C ≥ 0.91 mg/dL, Q4) independent of cardiovascular variables, while the risk was higher in late postmenopause than in early postmenopause. In addition, we observed that poor subjective sleep quality, shorter sleep duration (<6 h), low sleep efficiency (<75%) and delayed sleep latency and higher sleep disturbance showed increasing odds ratio for declining renal function in a dose-manner fashion.

Previous studies have supported that short sleep duration (<5 or 6 h), poor sleep quality and obstructive sleep apnea (OSA) are associated with a higher prevalence of CKD progression estimated by eGFR in patients with diabetic kidney disease, type 2 diabetes (16, 24–26). In addition, a 16.8 years of follow-up study demonstrated that both short and long sleep durations were associated with a higher risk of end-stage renal disease in Chinese population (27). However, little attention was paid to the menopausal women. The strength of our study was that we focused on the interaction of sleep characteristics and clinically latent renal disease in terms of menopause. Accordingly, we suggested that sleep disorder (both total PSQI score and sleep parameter) adversely impacted renal function independent of cardiovascular risk factors. Additionally, we indicated that longer years since menopause served as an incremental role of sleep disorder for declining renal function.

In addition, our multiple logistic regression analysis identified that a higher level of FSH was independently associated with declining renal function. In particular, participants in the fourth FSH were more likely to have renal dysfunction, which was in agreement with the results of the previous study that higher FSH was an independent risk factor declined eGFR and CKD in postmenopausal women (5). Of interest, we also found that MHT was an independent protective indicator associated with declining renal function. Our finding was consistent with the previous study that MHT can delay CKD progression and improved eGFR (28). Menopause is known to be a vital physiological stage of women’s lives characterized by increased FSH concentrations as a result of ovarian failure. Thus, our above results confirmed that the higher FSH level caused by menopause contributed to the worsening of renal function in women, whereas MHT may reversely attenuate the kidney injury. While the possible mechanism was that FSH promoted renal fibrosis in aging women *via* bonding to FSH receptor, which was expressed in kidney tissue (29, 30).

Other classical independent factors such as hypertension, diabetes, older age, BMI, postmenopause for declining renal function were compatible with previous studies (5, 29–31).

Although the underlying mechanisms by which sleep disorder induced declining renal function in menopause are not fully understood, the admissible mechanisms can be boiled into direct and indirect ways. Sleep disorder can lead to inflammation, oxidative stress, endothelial dysfunction, increased sympathetic tone, activation of the renin-angiotensin system, circadian timing dysfunction and subsequent systemic and intraglomerular pressure, which hereby adversely affects kidney function (24–26, 31, 32). Another possible explanation is the impact of sleep disorder on hypertension (33, 34), diabetes (35, 36), obesity (37), and metabolic syndrome (38), which were known to accelerate deterioration of kidney function.

Several limitations deserve mention in this research. First, our study is a cross-sectional analysis, the inherent drawback of an observational survey may weaken the causal relationship. Secondly, sleep quality ascertained by questionnaire would produce memory bias. Therefore, further longitudinal study is needed to confirm these relationships. Our team is now working on the following-up investigation.

## Conclusion

Taken together, our study demonstrated that both cumulative (PSQI total score) and separate sleep dimension (subjective sleep quality, shorter sleep duration, low sleep efficiency delayed sleep latency and higher sleep disturbance) were independently associated with declining renal function (the highest Cys-C, Q4) in postmenopausal women. Thus preclinical prevention such as MHT should be taken for postmenopausal women with sleep problems.

## Data availability statement

The raw data supporting the conclusions of this article will be made available by the authors, without undue reservation.

## Ethics statement

The study protocol was approved by the Ethics Committee of Shanghai Sixth People’s Hospital. The patients/participants provided their written informed consent to participate in this study.

## Author contributions

MT conceived and designed the study. YZ analyzed the data. JT wrote the manuscript. CL and JH took part in the investigation and data collection. YT revised the manuscript. All authors read and approved the final manuscript and contributed to the article and approved the submitted version.

## Abbreviations

CKD: chronic kidney disease
GFR: glomerular filtration rate
eGFR: estimated GFR
Cys-C: cystatin C
OSHS: obstructive sleep apnea-hypopnea syndrome
PSQI: Pittsburgh Sleep Quality Index
MHT: menopausal hormone replacement
STRAW: Stages of Reproductive Aging Workshop
TC: total cholesterol
TG: triglyceride
LDL-C: low-density lipoprotein cholesterol
HDL-C: high-density lipoprotein cholesterol
FSH: follicle-stimulating hormone
FBG: Fasting plasma glucose
MetS: metabolic syndrome
OSA: obstructive sleep apnea
CKD-EPI: Chronic Kidney Disease Epidemiology Collaboration.
